# LOCAL FAMILY AND FRIEND TIES AND THEIR RELATIONSHIP TO SOCIAL SUPPORT AND STRAIN AMONG OLDER ADULTS

**DOI:** 10.1093/geroni/igad104.1560

**Published:** 2023-12-21

**Authors:** Won Choi

**Affiliations:** University of Chicago, Chicago, Illinois, United States

## Abstract

Family members and friends who live nearby are likely valuable sources of support for older adults. At the same time, local family and friend ties may also be a source of strain as spatial proximity to close ties can generate more intense interactions. Using data from Round 3 (2015-2016) of the National Social Life, Health, and Aging Project (NSHAP) (N=3,615), this study examines how local family and friend ties reported in older adults’ social network roster are associated with instrumental and emotional support and social strain among community-dwelling older adults aged 50 and older. Results from ordered logistic regression models show that having a local friend tie is associated with higher levels of instrumental and emotional support from friends and lower levels of instrumental and emotional support from family. Having a local family tie, on the other hand, is associated with higher levels of instrumental support from family and lower levels of emotional support from friends. Having a local family tie is not related to emotional support from family or instrumental support from friends. Results also indicate that having a local friend tie increases the odds of reporting that friends make too many demands (i.e., higher friend strain) whereas having a local family tie is not a predictor of family strain. Together, results suggest that spatial proximity to friends and, to a lesser degree, family members are linked to how older adults experience social support and strain.

